# Effect of Probiotic Bacteria on Microbial Host Defense, Growth, and Immune Function in Human Immunodeficiency Virus Type-1 Infection 

**DOI:** 10.3390/nu3121042

**Published:** 2011-12-19

**Authors:** Susanna Cunningham-Rundles, Siv Ahrné, Rosemary Johann-Liang, Rachel Abuav, Ann-Margaret Dunn-Navarra, Claudia Grassey, Stig Bengmark, Joseph S. Cervia

**Affiliations:** 1 Weill-Cornell Cellular Immunology Laboratory, Division of Hematology/Oncology, Host Defenses Program, Department of Pediatrics, Weill Medical College of Cornell University (WCUMC), New York, NY 10065, USA; Email: abuavr@csmns.org; 2 Department of Food Technology, Lund University, Lund SE-221 00, Sweden; Email: Siv.Ahrne@appliednutrition.lth.se; 3 Division of Infectious Disease, Department of Pediatrics, Weill Medical College of Cornell University (WCUMC), New York, NY 10065, USA; Email: Rjohann-liang@hrsa.gov (R.J.-L.); ad66@columbia.edu (A.-M.D.-N.); cbgrassey@optonline.net (C.G.); Joe_Cervia@NSHS.edu (J.S.C.); 4 Division of Surgery and Interventional Science, University College London, 74 Huntley Street, London WC1E 6AU, UK; Email: s.bengmark@ucl.ac.uk

**Keywords:** microbial translocation, inflammation, probiotic bacteria, lactobacillus, HIV-1, AIDS, children, women, anti retroviral therapy, growth, failure-to-thrive, gut associated lymphoid tissue (GALT), mucosal barrier, microflora, CD4+ Th17 cells, CD4+ CD25+ FoxP3+ T regulatory cells, immune development, micronutrient, nutrition, body mass index (BMI), body cellular mass, BCM, anti-retroviral therapy (ART)

## Abstract

The hypothesis that probiotic administration protects the gut surface and could delay progression of Human Immunodeficiency Virus type1 (HIV-1) infection to the Acquired Immunodeficiency Syndrome (AIDS) was proposed in 1995. Over the last five years, new studies have clarified the significance of HIV-1 infection of the gut associated lymphoid tissue (GALT) for subsequent alterations in the microflora and breakdown of the gut mucosal barrier leading to pathogenesis and development of AIDS. Current studies show that loss of gut CD4+ Th17 cells, which differentiate in response to normal microflora, occurs early in HIV-1 disease. Microbial translocation and suppression of the T regulatory (Treg) cell response is associated with chronic immune activation and inflammation. Combinations of probiotic bacteria which upregulate Treg activation have shown promise in suppressing pro inflammatory immune response in models of autoimmunity including inflammatory bowel disease and provide a rationale for use of probiotics in HIV-1/AIDS. Disturbance of the microbiota early in HIV-1 infection leads to greater dominance of potential pathogens, reducing levels of bifidobacteria and lactobacillus species and increasing mucosal inflammation. The interaction of chronic or recurrent infections, and immune activation contributes to nutritional deficiencies that have lasting consequences especially in the HIV-1 infected child. While effective anti-retroviral therapy (ART) has enhanced survival, wasting is still an independent predictor of survival and a major presenting symptom. Congenital exposure to HIV-1 is a risk factor for growth delay in both infected and non-infected infants. Nutritional intervention after 6 months of age appears to be largely ineffective. A meta analysis of randomized, controlled clinical trials of infant formulae supplemented with *Bifidobacterium lactis* showed that weight gain was significantly greater in infants who received *B. lactis* compared to formula alone. Pilot studies have shown that probiotic bacteria given as a supplement have improved growth and protected against loss of CD4+ T cells. The recognition that normal bacterial flora prime neonatal immune response and that abnormal flora have a profound impact on metabolism has generated insight into potential mechanisms of gut dysfunction in many settings including HIV-1 infection. As discussed here, current and emerging studies support the concept that probiotic bacteria can provide specific benefit in HIV-1 infection. Probiotic bacteria have proven active against bacterial vaginosis in HIV-1 positive women and have enhanced growth in infants with congenital HIV-1 infection. Probiotic bacteria may stabilize CD4+ T cell numbers in HIV-1 infected children and are likely to have protective effects against inflammation and chronic immune activation of the gastrointestinal immune system.

## 1. Introduction

The original perception that intestinal mucosal starvation could be a central cause of the mucosal atrophy, opportunistic bacterial growth, and microbial translocation in untreated HIV-1 infection led to the hypothesis that gut reconditioning through probiotic administration could be protective for the gut surface and delay progression to AIDS [1]. An enteral formula was developed to generate a local probiotic effect in the lower gastrointestinal tract through fermentation of complex fibers and proteins. The combination of lactobacilli and fiber was expected to produce short-chain fatty acids and critical amino acids such as glutamine and arginine [1]. As discussed here, a number of studies have emerged that support the concept that probiotic bacteria can provide specific benefit in HIV-1 infection. The effects may be especially important in children who become infected before the development of a normal gut flora [2] and are at risk for chronic immune activation and growth abnormalities. 

Malnutrition including critical micronutrient deficiencies has been well recognized as a major co-factor in morbidity and progression to AIDS [3,4,5,6,7,8,9,10]. Early attempts to use nutrient supplementation showed benefit. Glutamine treatment reduced malabsorption and was subsequently included in the management of diarrhea in AIDS [3,11,12]. While nutrient support could not restore gut function, the use of complex regimens, in conjunction with optimal treatment of concurrent co-infections has achieved clinical goals [13]. Micronutrient supplementation has shown significant potential for treatment of HIV-1/AIDS excepting the enhancing effect of maternal Vitamin A supplementation on transmission of HIV-1 through breast milk [14,15]. 

Current studies do show that weight gain after initiation of effective anti retroviral therapy (ART) is associated with improved survival and decreased risk for clinical failure in adults [16]. Investigations into the mechanisms of HIV-1 associated growth problems in children have suggested that long term effects on height can not be explained solely by inadequate nutrition or endocrine abnormalities, and that deficits are not fully reversible by optimal anti-retroviral therapy. The combined interaction of gastrointestinal tract dysfunction, chronic or recurrent, infections, and chronic immune activation is likely to contribute to nutritional deficiencies and also to have specific effect on growth in the HIV-1 infected child [17]. 

The recognition that changes in the microbiota are associated with profound impact on metabolism has generated new investigations into potential mechanisms of gut dysfunction and inflammation in HIV-1 infection [18,19]. Fundamental experiments in germ-free mice demonstrated that the gut microbiota affect metabolism, and that altered states can lead to the development of inflammatory diseases [19]. Emerging studies show that HIV-1 disturbs the microbiota of the host early in infection leading to greater dominance of potential pathogens, reducing levels of bifidobacteria and lactobacillus species and increasing mucosal inflammation. Loss of the mucosal barrier and exposure to potentially opportunistic pathogens such as *Pseudomonas aeruginosa* and *Candida albicans* can alter the normal growth program through multiple effects [20]. Altered microbiota in several mucosal niches may promote host conditions known to be involved in enhanced transmission of HIV-1 disease such as bacterial vaginosis (BV) in HIV-1 positive women. Recent metagenomic studies have identified vaginal microbiota associated with BV in HIV-1 positive women at the genus level [21]. Other studies have shown that HIV-1 infection is associated with shifts in vaginal microflora characterized by reduced lactobacilli and increased number of taxa including Propionibacterineae, Citrobacter, and Anaerococcus that were not found in HIV-1 negative women [22]. As discussed below, probiotic bacteria promote normalization of flora in HIV-1 infected women with moderate bacterial vaginosis [23]. Another recent study shows that dietary supplementation with a prebiotic oligosaccharide mixture improved the gut microbiota composition, reducing biomarkers of microbial translocation and T cell activation [24]. The impact of HIV-1 on the development of the microflora in a newborn infant is likely have significant effects on growth and development of the local immune system, since both commensals and pathogens activate neonatal immune response [25,26,27,28]. Further studies are clearly needed. A recent experimental study has shown that disturbing the normal bacterial colonization after birth affected both gut growth and function [29]. We present results from a small pilot study indicating that administration of probiotic bacteria to children with HIV-1 associated failure-to-thrive, had effects on growth as well as on peripheral immune blood immune response that also support this concept. Future studies in this area should be based on expanded methods of determining the presence and function of bacterial species by genetic and metagenomic approaches. 

Translocation of microbial products is now recognized as a principal cause of the chronic systemic immune activation and inflammation that promotes progression of HIV-1 disease. The identification of the Th-17 pathway and the role of T regulatory cells provides a plausible mechanism of action and may be highly relevant for the potential exploration of probiotic bacteria in the setting of HIV disease in the future.

## 2. Impact of Nutrient Status on HIV-1 Infection

Weight loss is a frequent feature of untreated HIV-1 infection. Recognition of the wasting syndrome, known as Slim disease in Africa and inclusion of unexplained wasting as a criterion for AIDS by the Centers for Disease Control (CDC) demonstrates the significance of altered metabolism for clinical progression of HIV-1. Until treatments for HIV-1 associated co infections had evolved to the point of control, wasting could not be directly attributed to HIV [30,31]. The possible similarity between HIV-1 associated weight loss and protein calorie malnutrition (PCM), previously the major cause of acquired immune deficiency world-wide, stimulated numerous investigations that sought to characterize changes in body composition and identify specific nutrient deficiencies. Alterations in fluid balance affect determination of lean body or fat-free mass such as body mass index (BMI) as commonly measured by anthropometry. Therefore assessment of body cellular mass (BCM) by measuring depletion of potassium or nitrogen provided the first clear indication that wasting was an independent risk factor for survival after HIV-1 infection [13]. Shortened survival correlated with greater degrees of BCM depletion [32]. The malnutrition of AIDS includes disorders of food intake, nutrient absorption and intermediary metabolism. Nutrient malabsorption was commonly observed in association with systemic infection or inflammatory conditions before the advent of highly active anti retroviral therapy ART. Initial studies indicated that mucosal architecture and absorptive abilities were relatively normal in AIDS patients in the absence of co-infection suggesting that intestinal function might be sufficient to maintain adequate nutritional status in patients without small intestinal injury [13]. However later studies revealed that HIV-1 was prominent in the gastrointestinal tract during clinical latency and was capable of causing mucosal inflammation in the absence of other enteric pathogens [33,34]. Furthermore, studies after 1996 with patients on highly active ART showed that loss of BCM did often continue in patients with low viral load and whose immunologic function was largely restored [35]. Altered metabolism is considered an adaptation to the underlying illness that leads to the acute phase inflammatory response. Mediated by upregulation of tumor necrosis factor and interleukins 1 and 6 [36], the acute phase response leads to increased resting energy expenditure [37]. Regaining weight in HIV-1 infection, particularly muscle mass, requires a combination of effective antiretroviral therapy, treatment of opportunistic infections, consumption of a balanced diet, physical activity, mitigation of side effects, and may require addition of appetite stimulants and growth hormone [38]. Abnormalities of lipid and glucose metabolism associated with HIV-1 and the lipodystrophies associated with antiretroviral therapies may also produce clinical symptoms readily confused with HIV-1 wasting [39]. Current studies show that abnormal metabolism occurs early in HIV-1 disease and increases with progressive immune dysfunction. In HIV-1 positive patients with a detectable viral load and no intestinal malabsorption, nutritional status impairment is often due to hypermetabolism [40]. Lack of biomarkers that distinguish between intestinal opportunistic infections and HIV-1 related enteropathy have hampered progress in this area. Recently Crenn *et al.* have shown that citrulline, the metabolic product of glutamine, related amino acids, and arginine synthesized by small-bowel enterocytes is a good biomarker of enterocyte mass, villous atrophy, and function in HIV-1 disease and can discriminate between protease inhibitor-related toxic diarrhea and infectious enteropathy [41]. The beneficial effect of energy dense and micronutrient fortified supplements as an adjunct to anti retroviral therapy has been shown in wasted adults with AIDS. While short term supplementation increased BMI over 3 months, there was no lasting effect after supplementation was discontinued although anti retroviral therapy was continued and the BMI declined [42]. Critical micronutrient deficiencies that accompany macronutrient deficits in HIV-1 infection include low circulating levels of zinc [43], selenium [44], vitamin B-12 and fat-soluble micronutrients that are malabsorbed, such as vitamin E and vitamin A, and beta-carotene [45]. Repletion of zinc may be blocked by inflammation [46], a common condition in HIV-1 infection. Some of the micronutrient deficiencies found in circulating blood of HIV-1 patients were reported before the effects of physiologic stress on micronutrient distribution were fully appreciated. However, the overall relationship between micronutrient depletion and progression to AIDS has been firmly established [47,48]. Hepatitis C, a common co-infection in HIV-1 infected sub groups has been recently recognized as a separate cause of low serum micronutrients [49]. Nutritional status continues to be an important determinant of HIV-1 outcomes. Although low vitamin A (retinol), vitamin E (alpha-tocopherol), and selenium are uncommon in HIV-1 infected patients on ART while zinc deficiency is common. Increased zinc and selenium levels appear to be associated with improved virologic control [50]. One long-term (18-month) prospective randomized, controlled trial of zinc supplementation study in HIV-1 infected adults showed that zinc delayed immunological failure and decreased diarrhea over time although without any effect on viral load [51]. Vitamin D deficiency has recently been identified as highly prevalent among HIV-1 infected adults [52]. Resistance to HIV-1 may be mediated by a Vitamin D receptor polymorphism that mediates enhanced response to Vitamin D [53]. A study of HIV-1 infected men with normal BMI in medical care, who were characterized by three different dietary patterns: juice and soda; fast food and fruit drinks; and fruit, vegetable, and low-fat dairy products showed that those in the fruit, vegetable, and low-fat dairy cluster had the highest BMI and CD4+ T cell count [54]. The low-fat dairy cluster also had the highest levels of fiber, protein, and micronutrients. Protease inhibitors which often cause diarrhea interfere with absorption of B12 so that greater intake is required to maintain normal levels [55]. Selenium appears to have inhibitory activity against HIV-1 [56]. A double-blind, randomized, placebo-controlled trial showed that high dose yeast selenium blocked the increase in viral load in HIV-1 infected patients treated with ART and placebo compared to patients who received ART and selenium. Selenium also indirectly increased the CD4+ T cell level [57]. A meta analysis showed that although neither vitamin A nor beta-carotene supplementation in adults significantly reduced HIV-1 disease progression, vitamin A halved all-cause mortality in trials involving HIV-1 infected African children [14]. Multiple micronutrient supplements reduced morbidity and mortality in HIV-1 infected pregnant women and their offspring and improved early child growth in one large randomized controlled trial in Africa [14]. While the potential value of micronutrient repletion is clear, more studies are needed to address the benefit of micronutrient supplementation with ART in particular groups of HIV-1 infected persons. As discussed below, overall studies show that changes in the gut associated lymphoid tissue GALT leading to chronic immune activation are critical for the outcome of HIV-1 infection. The number of genes and level of gene expression in gut mucosal T cells associated with both lymphocyte activation and inflammatory stress responses such as RANTES, Toll-like receptor-1, MIP-4, CSF-1R, TNF receptor super family 11A, MCP-2, and prostaglandin E receptor-4) were substantially higher in HIV-1 positive patients with high viral load (HVL) than in long-term non-progressors (LTNP) [58]. This study suggested that active viral replication in HVL patients led to chronic lymphocyte activation and inflammation in GALT. In contrast to the HVL patients who showed depletion of CD4+ T cells in blood and in jejunal biopsies, LTNP patients maintained normal CD4+ T cell levels in both compartments. Surprisingly LTNP patients displayed significant dysregulation of genes associated with lipid metabolism and nutrient absorptive functions similar to the profile of HVL patients. The investigators reported that a broad range of genes involved in lipid and carbohydrate metabolism as well as genes mediating xenobiotic metabolism (CYP450 family) were similarly down-regulated in both LTNP and HVL patients. In contrast genes associated with amino acid metabolism were downregulated only in HVL patients. The data suggest that gastrointestinal complications including nutrient malabsorption occur independently of the level of viral suppression. In the SIV model, multiple growth factors as well as genes mediating intestinal epithelial (enterocyte) repair and regeneration have been shown to be dysregulated in GALT during infection [59]. 

## 3. Role of Microbial Translocation on Inflammation and HIV-1 Progression

The potential importance of host exposure to microbial products after breakdown of the gut mucosal barrier in HIV-1 disease for progression to AIDS has only recently become apparent. Indication of a possible causal link between inflammation, HIV-1 progression, and circulating levels of bacterial products was first reported in 1997 by Stein *et al.* in a study comparing the relative levels of urinary butyrate, a unique product of microbial metabolism and cytokines in HIV-1 positive patients with and without weight loss and normal controls. Both butyrate and interleukin (IL) 6 levels were elevated in HIV-1 positive patients with weight loss compared to HIV-1 infected patients without weight loss or compared to normal controls [60]. Determination of casual relationships was not possible at this time. Chronic immune activation, characterized by polyclonal B cell activation had been reported early in the HIV-1 epidemic [61] and subsequently the importance of activated T cells [62] and increased levels of circulating cytokines became apparent. However, immune activation was considered a result of failure to control viremia that by promoting functional disruption of the immune system would inevitably lead to progressive immune deficiency [63]. Although T cell activation had been shown to predict progression [62], the complex relationships between immune compartments and viral reservoirs limited determination of critical relationships. Subsequent studies revealed that CD4+ T cell depletion of gut lymphoid tissue is an early event in the pathogenesis of both human HIV-1 disease and after Simian Immunodeficiency Virus (SIV) infection of rhesus macaques [64,65]. Importantly, restoration of mucosal immunity was delayed even after effective antiretroviral therapy [65]. 

The etiological significance of early damage to gut- associated lymphoid tissue (GALT) for disease progression was not fully appreciated until 2006 when Brenchley *et al.* identified translocation of microbes or microbial products without overt bacteremia as a major cause of systemic immune activation in HIV-1 and SIV infection [66]. The investigators measured changes in plasma levels of lipopolysaccharide (LPS), a major component of gram-negative bacterial cell walls with strong immune stimulating activity that has been widely used as a marker for microbial translocation in other inflammatory conditions including inflammatory bowel disease. In HIV-1 infected and AIDS patients, LPS levels were equally increased. LPS levels could be transiently reduced in susceptible rhesus macaques after SIV infection by antibiotic treatment. Increased levels of soluble CD14 (sCD14), secreted by CD14+ monocyte/macrophages in response to LPS, were observed in early HIV-1 infection and were found to be higher in those who progressed to AIDS. Naturally occurring immunoglobulin-M (IgM), immunoglobulin-A (IgA) and immunoglobulin-G (IgG) antibodies to the LPS core oligosaccharide, endotoxin-core antibodies (EndoCAb), which bind to and clear LPS from the circulation were lower in acute, early infection compared to the control, uninfected population. Brenchly *et al.* reported a significant positive correlation between plasma LPS levels and frequency of circulating CD8 T cells with an activated CD38+ HLA-DR+ phenotype, previously shown by Giorgi *et al.* to predict mortality from HIV-1 infection [62]. Elite controllers, who maintained low or undetectable plasma viral loads without treatment and had a low level of immune activation [67], had higher levels of plasma LPS with higher levels of EndoCAb, but lower levels of sCD14 compared to HIV-1 progressors. Thus in elite controllers increased microbial translocation was counter balanced by increased neutralization of LPS, and ,combined with decreased systemic response to LPS, formed an effective barrier against chronic inflammation [66]. One of the most interesting observations made by Brenchley *et al.* was that responders to effective antiretroviral therapy (ART) showed partially suppressed microbial translocation. Others showed subsequently that response to ART could normalize T cell subsets and reduce oligoclonal T cell expansion but that levels of activated CD8+T cells remained increased [68]. 

The interaction between lentiviral infection and chronic immune activation was revealed in compelling experimental studies of SIV infection. Control of SIV infection in chronically SIV-infected sooty mangabeys, SIV’s natural host, was associated with low immune activation and less fibrosis compared to uncontrolled SIV infection of susceptible rhesus macaques [69]. Recent studies have shown that viral replication in the GALT and marked CD4 T-cell depletion in pathogenic SIV infection correlates with decreased expression levels of genes regulating epithelial barrier maintenance and digestive/metabolic functions and coincides with increased transcription of genes linked to immune activation and inflammation [70]. Studies in humanized mice have shown that disruption of the intestinal barrier with dextran sulfate in uninfected mice while sufficient to induce microbial translocation did not lead to elevation of LPS due to effective monocyte phagocytosis of LPS [71]. In contrast monocytes from HIV-1 infected mice could not clear LPS and this led to increased plasma levels of LPS. Monocyte activation in human HIV-1 infection may be controlled through interferon alpha [72]. 

A multivariate analysis of immune reconstitution after ART showed that faster recovery to a CD4+ T cell count of >500 cells/µL was significantly associated with lower pre-ART LPS levels, and higher pre-ART sCD14 levels [73]. Therefore lack of CD4 recovery in individuals in whom ART suppressed HIV-1 replication could be caused by immune activation associated with higher LPS levels driven by altered gut permeability [74]. A study in children has shown that microbial translocation is detectable by assessment of plasma LPS and sCD14 in healthy infants but resolves with age. In contrast LPS and soluble CD14 levels were elevated in all HIV-1-infected children and persisted even if CD4 T cells were fully reconstituted, virus was suppressed, and when lymphocyte activation was controlled by ART [75]. 

Overall these studies suggest that levels of microbial translocation must be matched by mechanisms that protect gut mucosal surfaces, and ability to clear the translocated microbial constituents, in order to counterbalance pathological immune activation following acute HIV-1 infection [66,70,76]. The critical interactions are regulated by transcription factors and other signaling molecules and, as described below, are likely to be mediated by regulatory T cells.

## 4. Balance of CD4+ Th17 and T Regulatory (Treg) Lymphocytes and HIV-1 Pathogenesis

Discovery that depletion of T helper (Th) 17 (Th17) cells from the gut in HIV-1 infection is associated with microbial translocation, chronic immune activation, and disease progression has revealed a critical new pathway that normally protects the integrity of the mucosal immune system in healthy persons but is damaged by HIV-1 disease [70,77,78]. CD4+ Th17 cells have been specifically identified as a sensitive, early target for HIV-1 infection. Normal commensal microbes in the gut appear to be essential for initiating Th17 cell differentiation [79]. Th17 cells localized in mucosal tissues produce IL-17A which then binds to its receptor on epithelial cells to attract neutrophils and macrophages via chemokine production and also IL-22 which induces antimicrobial peptides [80,81,82]. CD4+ Th17 cells share differentiation pathways with antigen-induced and CD4+ CD25+ FoxP3+ T regulatory (Treg) cells. 

In mouse spleen cells probiotic *Lactobacillus casei* induced interleukin (IL)-12 production by CD11b+ cells more strongly than pathogenic gram-positive and gram-negative bacteria and promoted the development of T helper type 1 (Th-1) cells leading to high levels of secretion of interferon (IFN)-gamma *in vitro* while slightly decreasing the IL-17 response to ovalbumin in cells from Peyers patches. Thus regulation is coordinated and involves compartmental differences [83]. In HIV-1 infected persons, CD4+ Th17 cells were lost from the gut but not bronchoalveolar lavage fluid. The cytokine responses of CD4+ Th17 cells were activated by extracellular bacteria and fungi but not by viral antigens [77]. Since primate species that were able to control SIV viral load, such as sooty mangabeys, maintained healthy frequencies of Th17 cells in the blood and GI tract, this was evidence that Th17 cells were protective against lentivirus pathogenesis [77,84]. Later studies showed a link between generalized Th17 depletion and failure to induce an increase in (Treg) cells after acute SIV infection in susceptible primates. In contrast, African green monkeys that controlled SIV infection maintained Th17 cells and showed an early, sustained increase in Tregs after acute infection [85]. Overall a balance between Th17 and Treg cells appears to support nonpathogenic SIV infection while a lower Th17/Treg ratio, high levels of immune activation, and generalized CD4+ T cell depletion characterize pathogenic SIV infection.

Treg cells express toll-like receptor 4 (TLR-4) and are activated by LPS. Neonatal Tregs can be activated *in vitro* by probiotic strains of lactobacilli isolated from human breast milk [86]. One study showed that *L. reuteri* and *L. casei*, but not *L. plantarum* bind to the C-type lectin DC-specific intercellular adhesion molecule 3-grabbing non-integrin (DC-SIGN) on dendritic cells to stimulate functionally activated Treg cells that suppressed proliferation *in vitro* [87]. Numbers of HIV-1 specific Treg cells and activity are increased in HIV-1 patients who show viral suppression but have persistently reduced CD4+ T cells and immune activation associated with increased plasma LPS after ART [74]. Profound loss of Th17 cells and reduction of CD161 CD4 cells, which may limit Th17 reconstitution in untreated HIV-1 infection, is associated with a gradual decline in Tregs, increased immune activation, and disease progression in blood product associated HIV-1 infection in hemophilia patients [88]. 

The balance between the Th17 and Treg cell lineages is generally recognized as an important immune regulatory mechanism determining host protection from autoimmunity as well as infection [89,90,91,92]. Two recent investigations show that a combination of probiotics could up-regulate Tregs and suppress progression in mouse models of autoimmune disease. Lavasani *et al.* [xref^93^] fed five different strains of lactobacillus to groups of C57BL/6 mice before immunizing mice with a synthetic peptide of myelin oligodendrocyte glycoprotein known to induce experimental autoimmune encephalomyelitis (EAE). Three of the lactobacillus strains prevented or delayed the onset of clinical EAE. Since activation of autoreactive CD4 T cells and their migration to into the central nervous system (CNS) is required for EAE development, they assessed proliferative responses *ex vivo* and CNS infiltration *in vivo*. They found decreased T cell proliferation to the immunizing peptide and reduced CNS infiltration and also reduced production of pro inflammatory Th-1 cytokines (TNF and interferon gamma (IFNg) production and increased Th-2 (IL-4, IL-10, and TGF-beta) compared to untreated mice. However, no monostrain was effective as a treatment for established EAE. When they combined the active strains and fed a mixture of three *Lactobacillus* strains, *L. paracasei* DSM 13434, *L. plantarum* DSM 15312 and DSM 15313 to mice with established EAE, suppression of EAE disease was observed. Suppressive activity correlated with IL-10 producing Tregs and led to attenuation of pro-inflammatory Th1 and Th17 cytokines followed by IL-10 induction in mesenteric lymphnodes, spleen and blood [93]. Others have shown that administration of *L. acidophilus*, *L. casei*, *L. reuteri*, *Bifidobacterium bifidium*, and *Streptococcus thermophilus* induced both T-cell and B-cell hypo responsiveness, down-regulated Th1, Th2, and Th17 cytokines without apoptosis and led to migration of Tregs to inflammatory regions in mouse models of experimental inflammatory bowel disease, atopic dermatitis and rheumatoid arthritis [94]. Probiotic strains appear to differ in their capacity to induce functionally active Treg cells from CD25− CD4+ cells [95].

The potential role of probiotic bacteria as modifiers of immune response to HIV-1 may include effects on microbial translocation and type of immune activation, as well as balance of Th17 and Treg pathways and should be the focus of future studies.

## 5. Effect of HIV-1 and Probiotics on Microflora and Microbiota

As described above, host exposure to microbial products after injury to the GI tract is a major driver of chronic immune activation after HIV-1 infection. Furthermore recent studies point to the emergence of an abnormal microbiota after HIV-1 infection that could directly challenge and compromise host immune response and metabolism. The composition of GI tract microbiota has been assessed by fluorescence *in situ* hybridization or quantitative real time PCR of fecal cells from healthy, asymptomatic HIV-1 positive, ART-naïve individuals and compared to the results of a control general population. Levels of *P. aeruginosa* and *C. albicans* were very high compared to controls while bifidobacteria and lactobacilli were reduced. Levels of fecal calprotectin, a protein secreted by neutrophils recruited to the intestinal lining, were much higher than in controls [20]. Compared to healthy men, the levels of all lactobacillus species were lower in HIV-1 infected men [96]. Others have shown that number of lactic acid bacteria present in the intestinal tract of children infected with HIV-1 was reduced compared to healthy children and may be virtually absent in the majority of them [2]. Since HIV infected children are often treated with antibiotics to prevent secondary infections or to treat diarrhea, lactic acid bacteria may be further reduced. Treatment with trimethoprim/sulphamethoxazole has been found to reduce a large number of lactic acid bacteria species normally present in the intestinal tract of healthy children [2]. 

Bacterial vaginosis (BV), a common condition that is associated with amniotic fluid infection and premature birth is characterized by discharge, fishy odor, and increased vaginal pH above 4.5. Microbiologically, BV is characterized by a shift in the vaginal flora from the dominant flora of *Lactobacillus* spp. to a mixed flora that includes *Gardnerella vaginalis*, *Bacteroides* spp., *Mobiluncus* spp., and *Mycoplasma hominis* and is scored by standardized criteria [97]. BV is common among HIV-1 infected women and is associated with a 3-fold increase in vertical transmission of HIV-1 [98]. Sha *et al.* reported that female genital-tract HIV-1 viral load correlated inversely with lactobacillus species and positively with BV and *Mycoplasma hominis* [99]. When effects on the diversity of genital microbiota in HIV-1 infected women with BV were compared to controls with and without BV, the results showed that HIV-1 positive women had a more diverse microbiota [100]. Current studies have identified several bacterial vaginosis-associated bacterium in HIV-1 infected women that are thought to increase HIV-1 shedding and also a species that in the context of *L.* *crispatus* absence was linked with high pH [101,102] Intravaginal probiotic treatment with *L.* *reuteri*combined with *L. rhamnosus* GR-1 led to effective cure in more patients compared to application of metronidazole gel alone [103]. A systematic review published in 2009 analyzing four of 17 randomized controlled trials that were deemed eligible for inclusion concluded that probiotics show promise in BV [104]. The trials compared probiotics with placebo, probiotics used in conjunction with conventional antibiotics compared with placebo or investigated probiotics alone compared with conventional antibiotics. The primary outcome measures were restoration of normal lactobacilli microbiota with eradication of BV microbiota and secondary outcomes included resolution of symptoms as reported by the patient and clinical cure as reported by the physician or investigator. Analysis of the odds ratio and confidence interval for individual studies was suggestive that oral metronidazole with oral probiotics and the probiotic/estriol regimen had beneficial effects but were not conclusive due to small sample size, disparate probiotic preparations and variations in study methodology [105,106]. Subsequent studies have shown that oral *L. rhamnosus* GR-1 and *L. reuteri* RC-1 given in a randomized, double-masked placebo-controlled trial did not enhance response to metronidazole. However treatment of HIV-1 infected women who had an aberrant microbiota was associated with increased probability of a normal vaginal flora for those with an intermediate flora and led to normalization of vaginal pH suggesting that this probiotic combination has potential for prevention [23]. Deep sequencing in HIV-1 positive women with BV has now revealed several profiles including lactobacillus species-dominating clusters characteristic of low pH, normal microbiota, or bacterial vaginosis. Treatment with metronidazole reduced diversity but seldom led to a lactobacillus dominated flora [21].

The study of mucosal microbiota in the context of HIV-1 infection is a complex process that will in future benefit from advanced genetic and metagenomic technology as well as better definition and characterization of the constituent cells of the specific ecosystem. For example, the discovery that macrophages in the vaginal mucosa are monocyte-like and are susceptible to HIV-1 infection while the intestinal mucosa macrophages are not is highly relevant to the different role of these compartments in pathogenesis. Recently investigators have performed an in-depth analysis of microbial species in the microbiota of the subgingival mucosa. Using DNA probes, Goncalves *et al.* reported that putative periodontal pathogens are more prevalent in the subgingival microbiota of HIV-1 seronegative patients with chronic periodontitis, whereas species not usually associated with periodontitis are detected in higher frequency in HIV-1 seropositive subjects receiving ART [107]. Another study reported previously unidentified pathogens, including opportunistic infections in subgingival mucosa in HIV-1 infected persons [108]. Although unique periodontal diseases occur in association with HIV-1 infection, previous studies had reported that subgingival bacterial flora were similar in HIV-1 infected and healthy with or without periodontal disease [109,110], despite lower numbers of bacterial species in saliva [111]. 

*Lactobacillus reuteri* was the first probiotic bacteria given to HIV-1 positive patients. The study proved that colonization could be achieved through oral administration although at a lower level than in healthy persons [96]. *L. reuteri* in combination with *Lactobacillus rhamnosus* GR-1 was effective as a therapy to combat diarrhea in HIV-1 positive women. In a small study of otherwise untreated HIV-1 positive women who were given a yogurt containing *Lactobacillus delbruekii* var. *bulgaricus* and *Streptococcus thermophilus*, the participants reported reduced diarrhea, flatulence, and nausea and showed stabilization of CD4+ T cell numbers [112]. 

The incidence and morbidity of HIV-1 infection is greatly increased in global regions of the world that also suffer from endemic diarrheal diseases. Since probiotic bacteria have proven effective in HIV-1 infected patients with other co-infections, the use of probiotic containing foods combined with treatment to replace fluids and electrolytes losses along with nutritional support has been proposed as an adjunct to specific anti-viral therapy [113]. One pilot study of a probiotic juice preparation given to HIV-1 infected children with failure-to thrive, showed beneficial effects on diarrhea and growth [7]. *In vitro* studies have shown that live probiotic bacteria protect epithelial cells from enteroinvasive Escherichia coli [114], a combination of *Streptococcus thermophilus* and *Lactobacillus acidophilus* ameliorated epithelial dysfunction due to inflammatory cytokines *in vitro* and in a mouse model of colitis *in vivo*[115]. Some probiotic bacteria are effective against infectious diarrhea in non-HIV-1 infected persons. *Lactobacillus rhamnosus* GG, LGG (ATCC 53103); shortened the duration of rotavirus diarrhea and enhanced humoral immune responses [116,117]. Salminen *et al.* examined the safety and efficacy of LGG in ameliorating prolonged gastrointestinal symptoms in HIV-1 patients on antiretroviral therapy. However no benefits were found against protease inhibitor-induced diarrhea induced by inhibitors in this small placebo-controlled, crossover study [118]. 

Another key area that has appeared promising for probiotic intervention is severe acute malnutrition in children. Diarrhea and malabsorption, small bowel overgrowth, increased intestinal permeability, enteropathy, gram-negative (enteric) bacteremia, and suboptimal immune response are common features in this presentation. While the results of large double masked placebo controlled clinical trial of prebiotics and probiotics with Synbiotic2000 Forte in an HIV-1 prevalent setting showed no direct benefits on outcomes related to malnutrition, the investigators did observe a possible reduction in mortality in the HIV-1 positive outpatient subgroup [119].

In underdeveloped countries with limited access to ART, the combined effects of malnutrition and opportunistic infections lead to high mortality. The gastrointestinal tract is the prime target. The digestive-absorptive functions are impaired with steatorrhea, nutrient malabsorption, and increased permeability occurs in 20-70% of children. Trois *et al.* used probiotics to assess benefits on immune response determined by CD4+ cells and on reduction of liquid stool episodes in a randomized double-masked controlled trial with 77 HIV-1 infected children (2-12 years). Children received a probiotic formula containing *Bifidobacterium bifidum* with *Streptococcus thermophilus* or a standard formula for 2 months. Mean CD4+ T cell count increased in the probiotic group while the mean CD4+ T cell count decreased in the control group over the same period. The incidence of loose-soft stools showed a small decrease in both groups [120]. A study in HIV-1 infected women used conventional yogurt fermented with *Lactobacillus delbruekii* var. *bulgaricus* and *Streptococcus thermophilus* that was supplemented or not with probiotic *Lactobacillus rhamnosus* GR-1 and *L. reuteri* RC-14. Anukam *et al.* reported that mean CD4+ T cell count remained the same or increased at 15 and 30 days in 11/12 probiotic-treated women compared to 3/12 in the control group. Diarrhea, flatulence, and nausea resolved in 12/12 probiotic-treated subjects within 2 days, compared to 2/12 controls receiving conventional yogurt for 15 days [112]. On balance these studies suggest that probiotics could enhance gut function and host defense against HIV-1 in the developing world. The benefit of probiotic bacteria may be especially important for children who have been congenitally exposed to HIV-1 for several reasons including provision of commensal microbes for priming the development of the gastrointestinal immune system, replacement for loss of lactobacilli, improvement of nutrient utilization, support of the gut barrier and the developmental program [121].

## 6. Growth Abnormalities in Children with HIV-1 Infection and Response to Probiotic *Lactobacilli*

Growth faltering in childhood is a nonspecific indicator of health problems and is associated with both short and long term adverse effects [122,123]. Poor growth and wasting are common manifestations of HIV-1 infection and AIDS in children worldwide [124]. The overall reported incidence of low birth weight and intrauterine growth retardation in a large birth cohort study in the mid 1990s was higher among children of seropositive mothers but [125] the mean birth weight of HIV-1 infected and uninfected children was not significantly different [126]. The interaction between nutrients, gut function, and HIV-1 viral infection is complex and remains to be clarified. In resource-poor countries, children born to HIV-1 infected mothers who were not infected have often shown poorer growth and higher morbidity and mortality than their unexposed peers. Catch-up growth in such children has varied across studies. The reasons for this are not clear and may involve fetal programming factors unique to the HIV-1 infected mother or poor maternal nutrient status. Whereas provision of a richly micronutrient-fortified diet given to non-breast fed children of HIV-1 infected mothers from 6 to 18 months improved hemoglobin and iron status and reduced stunting at 18 months, Filteau *et al.* reported that the same diet did not permit catch up growth in HIV-1 exposed, but uninfected Zambian children who received variable amounts of breast milk during the study [127]. The results suggest that early effects of nutrient deficiency may be irreversible at 6 months. One reason for this may be that poor nutrition impairs the development of a normal gut flora [127]. Infant milk source and supplementation are known to affect the composition of the microbiota [128]. In formula-fed infants, a complex intestinal microbiota develops with more coliforms, enterococci, bacteroides, and clostridia than in breast-fed infants. In contrast, Bifidobacterium species are usually predominant both in numbers and in frequency in fecal samples from breast-fed infants. The changing composition of the intestinal is caused by changes in nutrition during the first year of life. A current clinical investigation involves the administration of concentrated formula to infants born to HIV-1 infected mothers beginning at birth to determine if early provision of nutrient support would be more effective. Winter *et al.* have reported initial evidence of weight gain at eight weeks in uninfected infants [17]. This approach would also support the early development of the mucosal immune system which is dependent upon nutrient status [129,130,131] and provide proof of principle that HIV-1 infected infants should given nutrient support before 6 months. The potential added benefit of probiotic bacteria could also be considered in future. Steenhout *et al.* have carried out a meta analysis of 5 randomized controlled clinical trials in which a large number of infants received formulas containing a probiotic *Bifidobacterium lactis* (CNCM I-3446) and a sub-analysis in infants of HIV-1 infected mothers. Growth measurements (weight and BMI) from enrollment to 120 days were compared between infants fed a formula containing *B. lactis* and those fed a control formula. Infants whose mothers were not HIV-1 infected grew equally well on both formulae while among infants with HIV-1 positive mothers, weight gain of those taking *B. lactis* was significantly higher than of those not taking *B. lactis* by 3.1 g/day and the BMI gains were significantly higher as well [132]. 

While ART has enhanced survival in HIV-1 infected children, severe wasting is still an independent predictor of survival [133]. Stunting in particular may not resolve with the administration of ART [134] and even when sustained growth response does occur during ART therapy, normal levels are not attained [135]. The pathophysiology of growth abnormalities is incompletely understood, although evidence has accumulated showing that HIV-1 infected children have growth hormone (GH) resistance relative to HIV-1 uninfected children [136]. Lower insulin-like growth factor-1 (IGF-1) and IGF-1 binding protein-3 (IGFBP-3) levels were observed in HIV-1 infected children while IGFBP-1 levels were higher when growth was impaired [137]. Initiation or change in ART led to increased muscle mass and IGF-1 but not linear growth [137]. Recent data in a mouse model of immune deficiency showed that compromise of intestinal homeostasis leads to inflammation and wasting and is driven by altered interaction between the microbiota and GALT development [138].

Although growth delays resolve more readily in developed countries [139] growth abnormalities are a common AIDS defining condition for children in the United States [140]. Several types of disturbed growth patterns have been described, ranging from symmetric delays in weight and length or height to severe wasting with normal length or height. In developed countries, both weight and length or height decline in infected children as early as the first month of life tends to remain below that of exposed but uninfected children although dietary intakes are adequate [124]. A recent multivariate analysis has shown that factors predicting HIV-1 associated failure-to-thrive (FTT) in children born to HIV-positive mothers include history of pneumonia, maternal illicit drug use during pregnancy, lower infant CD4 T-cell count, exposure to antiretroviral therapy by 3 months of age (non-protease inhibitor), and HIV-1 RNA viral load [141].

The analysis of growth in pediatric anti-HIV-1 clinical trials plays an important role in trial evaluation. Growth failure may be a manifestation of progressive disease or treatment toxicity, and is commonly specified as a major trial outcome event indicating poor treatment performance. The relationship between viral load reductions and achievement of a favorable somatic growth profile are uncertain and new criteria to measure growth have been proposed [142]. This analysis showed that patients whose weight or height growth velocities remain below the 10th percentile of context reference distributions for more than 2 months were at significantly increased risk of death, controlling for sex, age and baseline immunologic status [142]. 

## 7. Effect of Oral Administration of *Lactobacillus* in HIV-1 Infected Children with Failure-to Thrive (FTT)

We have carried out a small pilot study using oral administration of a probiotic bacterium, *Lactobacillus plantarum* 299v, (Lp299v) to influence growth and immune development in children congenitally exposed to HIV-1 who were diagnosed with FTT. The observations presented here are relevant to the potential effects of probiotic bacteria in HIV-1 infected children in resource-poor countries as described previously [7,125,143]. The patient population was diagnosed with FTT and had received optimal antiretroviral therapy for at least one month prior to enrollment and had normal for age hematological, renal, and hepatic profiles. There were 10 girls and 4 boys. Ages ranged from 11.5 months to 14 years (mean age was 6.9 years). Twelve of the HIV-1 positive children were characterized as having FTT and were less than 5% for age adjusted height and/or weight at the time of study. An additional HIV-1 positive child who had normal weight and height but showed decreased velocity of growth for head circumference was also enrolled. Children or their parents chose between two identically labeled packets containing either Lp299v, or placebo prepared as a lyophilized powder in an oatmeal base in 5 gram amounts. Colonization was directly tested by culture of rectal swabs onto Rogosa agar plates, a medium selective for lactobacilli. Colonies were genetically typed for presence of Lp299v. No child was colonized with Lp299v prior to oral administration and all children given active Lp299v became colonized. There were no adverse side effects; colonization was temporary and was not maintained in the absence of continued treatment.

Ten children were evaluated for changes in height and four children were considered inevaluable for several reasons: one child was unable to stand and height measurements were not reliable, three children had either inadequate pre treatment or post treatment evaluation. Unblinding revealed that only one child did not receive active product.

**Figure 1:**
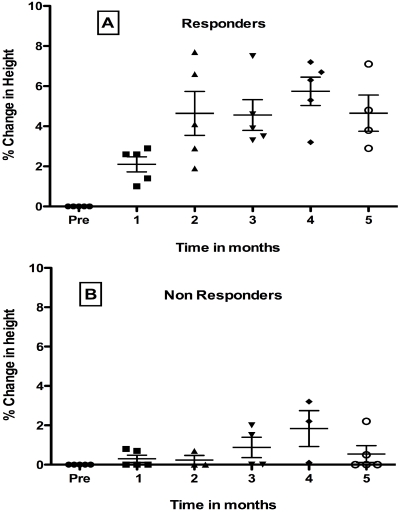
Effect of Oral Probiotic Lp299v on Height in Congenital HIV-1 Infection. Data show mean percent change ± SD. from mean baseline height for the 4-month pretreatment period compared to each post treatment month. For responders (panel A) but not non-responders (panel B), variance was significant. Mean height increased in post months 2, 3, 5 (*p* < 0.001), and 4 (*p* < 0.0001) by Tukey’s post test. See text for details.

As shown in Figure 1, overall variance was significant for responders in Panel A but not for non-responders in Panel B (*p* < 0.0001). Of nine patients who received active LP 299v, five showed a significant improvement of 5% or greater in height when heights during the four pretreatment months (-3, -2, -1 month, and Day 0) were compared to heights during five post treatment months.

Tukey’s post-test analysis showed that percent increase in height was significant for responders (panel A) at post months 2, 3, 5 (*p* < 0.001), and 4 (*p* < 0.0001) compared to the pretreatment period, but were not yet significant at the first post treatment month. In contrast, for non-responders shown in panel B, overall variance was not significant by 1-way ANOVA.

**Figure 2:**
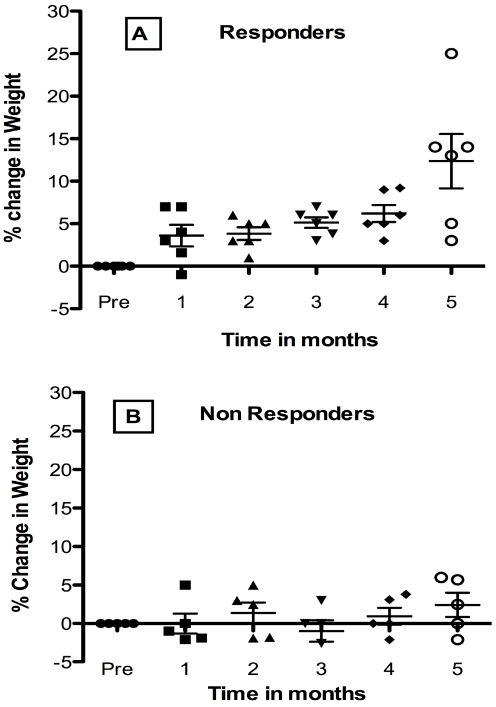
Effect of Oral Probiotic Lp299v on Weight in Congenital HIV-1 Infection. Data show mean percent change ± SD from the 4-month pretreatment period compared to post-treatment months. Variance was significant for responders (panel A). Responder pair-wise differences at post-month 5 compared to pretreatment (*p* < 0.0001) months were found. See text.

Changes in weight after Lp299v supplementation were also studied. Responders and non-responders were classified according to increase equal to or greater than 5%. The analysis of changes in weight was carried out as described for height described above, by comparing the average of 4 pretreatment weights to post-month treatment changes. Six of 11 evaluable children were responders as shown in Figure 2 panel A. 

Differences between pretreatment weight and percent change in weight post treatment were significant at post-month 5 compared to pretreatment (*p* < 0.0001) by Tukey’s post ANOVA analysis. Pair-wise comparisons between later post-months and early months were significant, including post month 5 compared to post-month 1 (*p* < 0.0001), or post month 2 (*p* < 0.001). Among the non-responder group overall differences among pre and post treatment weights were not significant by 1-way ANOVA. Examination of Figure 2, suggests that this was probably due to greater recurrent weight loss in the non-responders. Changes were not made to anti HIV-1 therapy for any child during the study period. Growth improvement occurred without reduction in viral load. Further study of the responders in comparison to the non-responders showed that the non-responders were on average older with a mean age of 10 years compared to responders whose average age was about 4 years of age. The younger responders also had a higher viral load. Overall the study indicates the potential benefit of probiotic supplementation in children with AIDS who have growth abnormalities.

**Figure 3:**
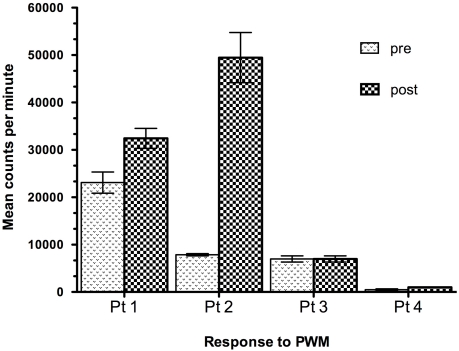
Response to Mitogen after Oral Probiotic Lp299v Treatment in Congenital HIV Infection. Data show pre-treatment compared to post-treatment proliferative response to pokeweed mitogen (PWM) *in vitro* at 3 months for 4 representative patients who were given oral Lp299v supplements. Mononuclear cells were cultured in triplicate and plated at 50,000 lymphocytes per well and pulse labeled with radio labeled tritiated thymidine. Data are shown as maximum mean response to a range of doses after 4 days total culture.

The effect of Lp299v on peripheral blood immune response was also studied *in vitro* by lymphocyte proliferation assay. At baseline, HIV-1 positive children had variable response to pokeweed mitogen ranging from normal to highly reduced, compared to normal controls. At 3 months post treatment some children who received active probiotic lactobacillus showed enhanced response, but not all. Improved immune functional response was independent of growth response. Typical patterns are shown for 4 patients in Figure 3.

Lp299v supplements did not enhance CD4+ T cell numbers in the group as a whole or lead to time dependent or consistent effects on relative or absolute CD4+ numbers of T lymphocytes. We previously reported the results of a study looking at the association between CD4+ T lymphocyte percentage and functional T lymphocyte immune response as assessed in the lymphocyte proliferation assay in HIV+ children with failure to thrive. When the children were grouped into those who had a higher average percentage of CD4+ T cells (30%) and compared to those with a lower percentage (7%), we found that both groups had equivalent and highly reduced lymphocyte proliferative responses to mitogen [144]. Others have shown that microbial translocation is not fully controlled by antiviral therapy and is associated with inefficient CD4+ reconstitution, A recent study showed that adults with advanced AIDS who were partial responders or non responders to ART had similarly elevated plasma levels of LPS and its ligand sCD14 that were not lowered by virologically suppressive therapy. Furthermore the investigators detected a highly polymicrobial peripheral blood microbiota both prior and after 12-month ART. Several differences in bacterial composition were shown between patients’ groups, mainly the lack of probiotic lactobacilli both prior and after therapy in the immunological non responders [145]. The use of newer methods to characterize the circulating microbiota in patients with HIV-1 infection is needed to elucidate the potential role of probiotic bacteria in reducing inflammation and in improving immune function.

## 8. Summary

Emerging information clarifying the role of GALT, altered microflora, and breakdown of the gut mucosal barrier in the etiology of HIV-1 disease have strengthened the rationale for use of probiotic bacteria in this setting. The recognition that changes in the normal bacterial flora have a profound impact on metabolism and inflammation has generated insight into potential mechanisms of gut dysfunction in HIV-1 infection. Current studies show that loss of gut CD4+ Th17 cells that differentiate in response to normal microflora, occurs early in HIV-1 disease. Microbial translocation and suppression of the Treg cell response are associated with chronic immune activation and inflammation. The balance between CD4+ Th17/Treg lymphocytes comprises a critical determinant of pathogenesis in both HIV-1 and SIV lentiviral infections. Before wide spread experience with effective anti retroviral therapy, chronic immune activation was considered the result of residual failure of less powerful anti viral regimens to control viremia. New studies show that combinations of probiotic bacteria can upregulate Treg activation sufficient to suppress pro inflammatory immune response in models of autoimmune diseases including inflammatory bowel disease, suggesting a mechanistic basis for use of probiotics in HIV-1 in combination with ART.

Disturbance of the microbiota early in HIV-1 infection leads to greater dominance of potential pathogens, reducing levels of bifidobacteria and lactobacillus species and increasing mucosal inflammation. Loss of lactobacillus species is characteristic of HIV-1 infection and is also caused by antibiotic treatment. Probiotic treatment leads to colonization of the HIV-1 infected host and can promote the restoration of normal vaginal flora in women with bacterial vaginosis, which could be protective against HIV-1 transmission. Combined analysis of four randomized placebo controlled trials has concluded that probiotics given as a metronidazole/probiotic regimen and probiotic/estriol show promise of effectiveness. Probiotic bacteria have been studied for potential amelioration of morbidity of diarrheal diseases, since some probiotics have proven effective against infectious diarrhea. A combination of several probiotic bacteria was effective in HIV-1 positive women in reducing symptoms and also led to stabilization of CD4+ T cell numbers. A study in children who received a probiotic formula or conventional formula showed a similar improvement in symptoms of diarrhea on both formulas. However children receiving the probiotic formula showed an increase in the mean CD4+ T cell counts while mean CD4+ T cell counts decrease in the control group on conventional formula suggesting specific effects on the immune system. 

The combined interaction of gastrointestinal tract dysfunction, chronic or recurrent, infections, and chronic immune activation is likely to contribute to nutritional deficiencies and also to have specific negative effects on growth In the HIV-1 infected child. Experimental studies show that disturbance of normal bacterial colonization after birth affects gut growth and function. Furthermore normal breast milk contains probiotic strains of lactobacilli that activate Treg differentiation that may be unavailable to the congenitally HIV-1 exposed child. While ART has enhanced survival in HIV-1 infected children, severe wasting is still an independent predictor of survival and a major presenting symptom. A meta analysis of randomized, controlled clinical trials of infant formula containing *Bifidobacterium lactis* showed significant effects on weight gain. We have observed that oral administration of a probiotic bacteria, *Lactobacillus plantarum* 299v, (Lp299v) influenced growth and immune development in children congenitally exposed to HIV-1 who were characterized as having failure-to-thrive. 

In summary current and emerging studies support the concept that probiotic bacteria can provide specific benefit in HIV-1 infection especially in women with bacterial vaginosis and in children who become infected before the development of a normal gut flora and are at risk for chronic immune activation and growth abnormalities.
